# RETRACTION: The Use of Medium‐Deep Peelings to Treat Melasma: A Case Series Study

**DOI:** 10.1111/jocd.70498

**Published:** 2025-10-09

**RETRACTION**: M. T. Scardua , N. Scardua , M. F. Ismail , W. Bambirra Junior , I. M. Candeias Carpinteiro , A. L. Oliveira Costa , and A. L. Sena Guimarães , “The Use of Medium‐Deep Peelings to Treat Melasma: A Case Series Study,” Journal of Cosmetic Dermatology 24, no. 8 (2025): e70291. 10.1111/jocd.70291.40820687 PMC12358819

The above article, published online on 18 August 2025 in Wiley Online Library (wileyonlinelibrary.com), has been retracted by agreement between the journal Editor‐in‐Chief, Michael H. Gold; and Wiley Periodicals LLC. Multiple third parties reported concerns that the research involves phenol‐based products, the use of which has been prohibited in Brazil for general health or aesthetic procedures following a resolution by Brazil's National Health Surveillance Agency (Anvisa) in June 2024 [1]. The research was conducted in Brazil, raising concern about the use of banned products in the study. The third parties expressed concerns that the article validates the use of phenol‐croton oil peel by non‐medical professionals and that the article does not declare a financial conflict of interest for several of the authors. The authors supplied a substantial response to these concerns, including copies of ethical approval for the retrospective case series from Universidade Estadual de Montes Claros, and documents concerning trademarks held by author M. T. Scardua. The authors declared that the study received proper ethical approval, that all procedures followed Brazil's regulations, and that the study was unrelated to trademarks and branding owned by some authors, therefore no conflict of interest statement was required.

The editor and publisher have determined that, according to the reported treatments presented in Supporting Information File S2, no treatments using phenol‐croton peel were provided following Anvisa's prohibition in June 2024. After June 2024, all treatments were reported as having used trichloroacetic acid (TCA), which is not under prohibition by Anvisa. However, the above parties have expressed concerns that Anvisa's June 2024 resolution was not mentioned in the article. Additionally, due to the prohibition of phenol‐croton products in Brazil, the treatments of several patients may have been changed from phenol‐croton to TCA, which may have affected the interpretation of the methodology and results.

Crucially, the above parties also determined that author M. T. Scardua holds a trademark for related phenol‐containing treatments and these treatments have been commercialized and promoted by some authors. These findings constitute financial interests that represent a conflict of interest, according to the policies of the publisher and the Committee on Publication Ethics (COPE). The editors determined that, had these financial interests been declared upon submission, they would have negatively affected the editors’ initial decision to have the manuscript peer reviewed.

The retraction has been agreed to because the editors have determined that the undeclared conflict of interest would have significantly affected their decision to consider the submission to the journal. As such, they no longer have confidence in the results presented in the article. The authors disagree with the retraction.

**Reference**

[1] National Health Surveillance Agency ‐ Anvisa . “Anvisa prohibits sale and use of phenol‐based products in general or aesthetic health procedures.” Last modified June 06, 2024. Translated by Google Translate. https://www.gov.br/anvisa/pt‐br/assuntos/noticias‐anvisa/2024/anvisa‐proibe‐venda‐e‐uso‐de‐produtos‐a‐base‐de‐fenol‐em‐procedimentos‐de‐saude‐em‐geral‐ou‐esteticos.

